# The efficacy and safety of danuglipron and orforglipron in patients with type 2 diabetes and obesity: a systematic review and meta-analysis

**DOI:** 10.3389/fendo.2025.1646956

**Published:** 2025-12-10

**Authors:** Jingjing Zhou, Fang Wang, Sen Li

**Affiliations:** 1 Department of Epidemiology and Health Statistics, Shanxi University of Medicine, Fenyang, China; 2 Department of Endocrinology, Fenyang Hospital, Fenyang, Shanxi, China; 3 Department of Health Information, Shanxi University of Medicine, Fenyang, China

**Keywords:** efficacy, danuglipron, orforglipron, type 2 diabetes and obesity, meta-analysis

## Abstract

**Objective:**

This study assesses the efficacy and safety of the novel oral small molecule glucagon-like peptide-1 receptor agonists (GLP-1 RAs) danuglipron and orforglipron in the treatment of type 2 diabetes (T2DM) and obesity through systematic review and meta-analysis.

**Methods:**

Electronic databases (PubMed, Web of Science, Cochrane Library and Embase) were systematically searched up to 20 May 2025 to include randomised controlled trials evaluating danuglipron/orforglipron in patients with T2DM and/or obesity. Changes in glycated haemoglobin (HbA1c), fasting plasma glucose (FPG), fasting plasma insulin (FPI), weight and body mass index (BMI) compared with baseline post-treatment were evaluated using random-/fixed-effects models, alongside safety outcomes.

**Results:**

Eight studies with low bias risk involving 1,454 participants were analysed. Meta-analysis results demonstrated that danuglipron significantly decreased HbA1c (mean difference [MD]: −0.90; 95% CI: −1.06, −0.74), FPG (MD: −24.66; 95% CI: −30.45, −18.86) and weight (MD: −2.17; 95% CI: −3.10, −1.23) and improved FPI (MD: 2.94; 95% CI: 1.50, 4.38). Orforglipron also showed significant positive effects on HbA1c (MD: −1.02; 95% CI: −1.18, −0.86), FPG (MD: −26.91; 95% CI: −31.05, −22.78), weight (MD: −6.28; 95% CI: −8.45, −4.11) and BMI (MD: −2.64; 95% CI: −3.38, −1.89). However, both danuglipron and orforglipron were associated with the occurrence of treatment-related adverse events and gastrointestinal adverse events (AEs).

**Conclusion:**

The oral GLP-1 RAs danuglipron and orforglipron are capable of improving blood glucose levels and reducing weight; however, they also pose an increased risk of gastrointestinal AEs. Further longitudinal studies are warranted to gain a deeper understanding of their efficacy, safety and tolerability.

## 1 Introduction

Diabetes (DM) is a global public health concern, and recent research indicates a substantial increase in the burden of the condition over the past decade, evolving into a growing epidemic. Specifically, 8.8% of adults are diagnosed with DM (1). If these trends persist, it is projected that by 2040, approximately 693 million individuals aged 18–99, comprising 9.9% of the world’s population, will have DM (1). The occurrence and progression of type 2 diabetes (T2DM) are closely linked to various modifiable risk factors (2). Among these factors, being overweight (body mass index [BMI] ≥ 25 kg/m^2^) and obesity (BMI ≥ 30 kg/m^2^) are the major influencers (3, 4). Additionally, limited physical activity, sedentary behaviour and certain habits (including high-calorie intake and smoking) contribute to the rising prevalence of T2DM (5, 6). In the clinical guidelines for managing T2DM without insulin use, recommended approaches include dietary adjustments and increased physical activity. If target blood glucose levels are not achieved, escalation to oral hypoglycaemic agents such as metformin or dipeptidyl peptidase-4 inhibitors is typically initiated (7).

Glucagon-like peptide-1 receptor agonists (GLP-1 RAs) provide a new avenue for treating T2DM. These agents activate the glucagon-like peptide-1 receptor (GLP-1 R), stimulating insulin secretion in a glucose-dependent manner and inhibiting glucagon secretion, thereby lowering blood sugar (8). Currently, several GLP-1 RAs are approved for treating T2DM, including liraglutide and semaglutide, known for their excellent glucose-lowering effects while also reducing body weight and blood pressure. However, their administration via subcutaneous injections may impact patient compliance. In comparison, patients may prefer and be more likely to adhere to an oral medication regimen. Presently, semaglutide is the only oral GLP-1 RA approved by the US Food and Drug Administration for T2DM treatment. However, patients taking oral semaglutide must do so at least 30 minutes before the first meal, drink or other medications of the day, limiting water intake to approximately 120 mL and avoiding food or drink for at least 30 minutes post-dose to ensure optimal absorption and efficacy (9). These restrictive intake conditions may affect patient compliance, highlighting the importance of seeking simpler oral GLP-1 RAs as a key treatment goal.

Danuglipron and orforglipron are both small molecule oral formulations of GLP-1 RAs, currently under development for treating T2DM and obesity. The recommendations are twice-daily oral intake for danuglipron and once-daily dosing for orforglipron. Due to orforglipron’s stronger impact on cyclic adenosine monophosphate (cAMP) signalling compared with beta (β)-cell recruitment, its lower risk of receptor desensitisation distinguishes it from other GLP-1 RAs (10). Recent clinical studies have explored the efficacy and safety of these two oral GLP-1 RAs, yet comprehensive evidence for their effectiveness and safety is lacking. Therefore, this study investigates the efficacy and safety of danuglipron and orforglipron in treating T2DM through meta-analysis, providing valuable insights for their clinical application.

## 2 Materials and methods

### 2.1 Search strategy

Following the PRISMA 2020 statement (11), a systematic search was conducted across four electronic databases: PubMed, Web of Science, Cochrane Library and Embase. The search period extended from database inception to 20 May 2025. Key search terms included ‘Danuglipron’, ‘Orforglipron’, ‘type 2 diabetes’, ‘diabetes’ and ‘obesity’. Additionally, relevant literature was obtained by reviewing the references of included studies.

### 2.2 Inclusion and exclusion criteria

The inclusion criteria were as follows: (1) randomised controlled trials (RCTs) published in peer-reviewed journals in both Chinese and English; (2) study participants with T2DM and/or obesity; (3) the intervention was the use of danuglipron or orforglipron; (4) inclusion of a control group receiving a placebo or blank control; (5) study outcomes included changes in glycated haemoglobin (HbA1c), fasting plasma glucose (FPG), fasting plasma insulin (FPI), weight and body mass index (BMI) compared with baseline; and (6) adverse events (AEs) included treatment-emergent adverse events (TEAEs) and gastrointestinal AEs. The exclusion criteria included (1) non-population studies; (2) study types such as conference articles, case reports and systematic reviews; (3) inadequate outcome information preventing data analysis; (4) duplicate publications; and (5) studies where complete articles could not be obtained.

### 2.3 Study selection and data extraction

The literature screening and data extraction process involved two researchers independently applying the inclusion and exclusion criteria. Initially, they conducted a preliminary screening based on reading the titles and abstracts of the literature, followed by a full-text review of studies that potentially met the inclusion criteria. In cases of disagreement between the two researchers, a third researcher’s opinion was sought for discussion to reach a consensus. After completing the literature screening, the two researchers independently performed data extraction using predefined data extraction forms. The extracted information included details about the literature, baseline demographic characteristics of the study participants, intervention protocols for danuglipron and orforglipron, study duration and outcome events.

### 2.4 Quality assessment

The Cochrane Collaboration’s risk assessment tool (12) was utilised to evaluate the quality of the literature. This method assesses aspects such as randomisation methods, allocation concealment, blinding, completeness of outcome data, selective reporting of study results and other sources of bias to ensure a comprehensive quality evaluation.

### 2.5 Analysis methods

Data analysis was performed using RevMan 5.3 (Cochrane, Northern Europe). Continuous outcomes were expressed as mean differences (MDs), whereas dichotomous outcomes were analysed using risk ratios (RRs). For studies with zero events (13), risk differences (RDs) were utilised for meta-analysis, with 95% confidence intervals (CIs) estimating the range of effect sizes. Heterogeneity was assessed using the *I*² statistic and Cochran’s *Q* test. If *I*² < 50% or *P* > 0.1, studies were considered homogeneous, and a fixed-effects model was applied; if *I*² ≥ 50% or *P* ≤ 0.1, significant heterogeneity was assumed, and a random-effects model was employed. When substantial heterogeneity was detected, sensitivity analyses were performed to explore its sources. Unless otherwise specified, a significance level of 0.05 was adopted for all statistical tests.

## 3 Results

### 3.1 Basic characteristics and quality of included studies

**Figure 1:**
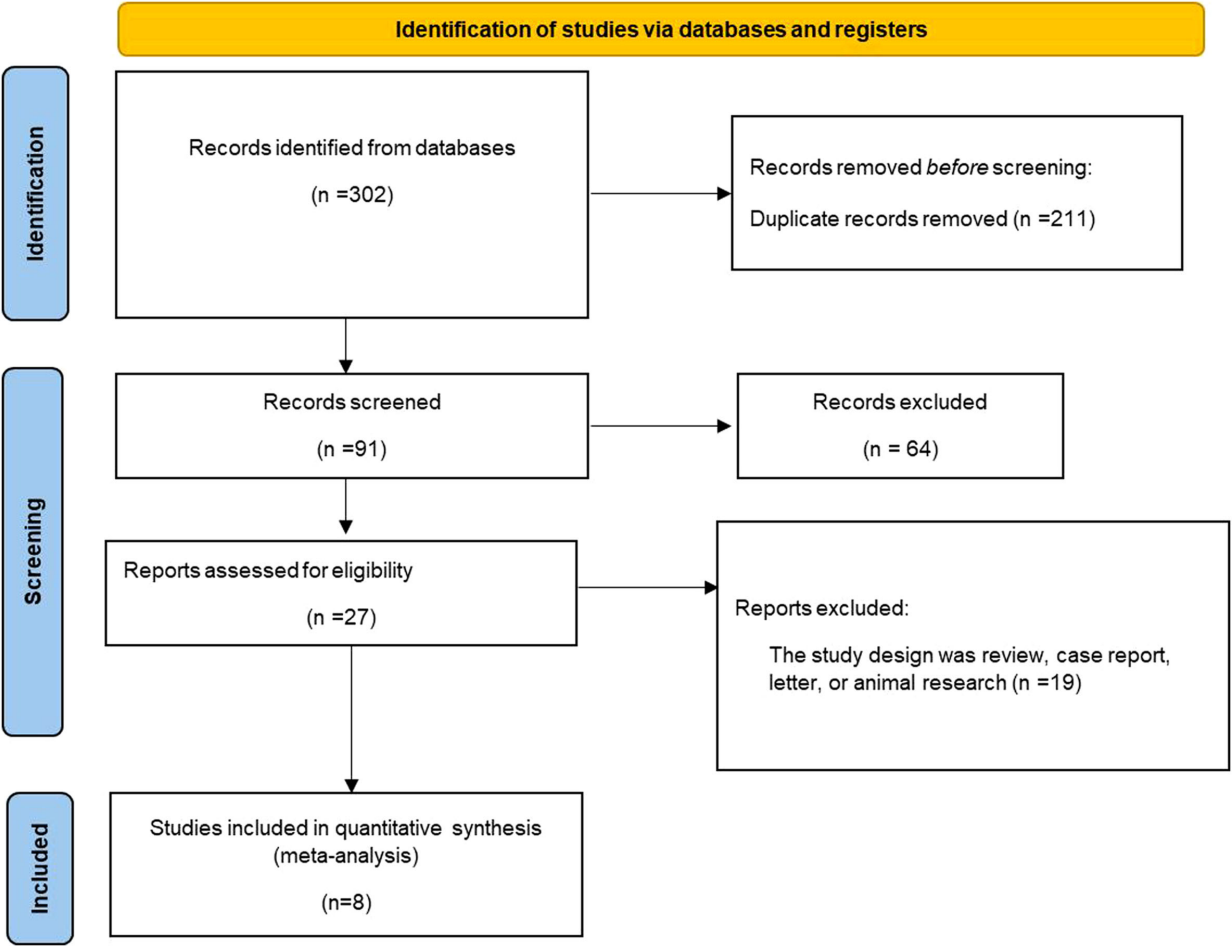
Flowchart of study selection.

Following systematic database searches, 302 studies were initially identified for screening. After excluding duplicates and irrelevant studies, 27 articles underwent full-text review. Ultimately, eight RCTs (14–21) met the inclusion criteria. The study selection process is detailed in **Figure 1**.

The baseline characteristics of the included studies are summarised in **Table 1**. All eight trials were published between 2021 and 2024, with seven being multicentre studies. All trials employed a double-blind design. A total of 1,454 participants were enrolled, with ages ranging from 42.5 to 62.8 years, male proportions varying between 27.3% and 88.9% and baseline BMI values of 25.9–38.7 kg/m². Five studies evaluated danuglipron at doses of 1.5–200 mg (twice daily), and four trials investigated orforglipron at doses of 2–45 mg (once daily).

**Table 1:** Basic information of included studies.

Study	Study design	Patients	Intervention	Sample	Mean age	male%	Disease duration, years	Weight, kg	BMI, kg/m2	Baseline HbA1C, %	Baseline FPG, mg/dl	Treatment duration
Saxena,2021	RCT, multicentre, double-blind	type 2 diabetes, obesity	Danuglipron, 10 mg bid	9	54.0 ± 6.22	77.8	11.7 ± 6.1	99.6 ± 12.7	33.9 ± 3.4	8.2 ± 0.6	158.3 ± 23.3	28 days
			Danuglipron, 15 mg bid	9	56.1 ± 9.53	33.3	8.1 ± 4.2	92.9 ± 22.5	35.3 ± 5.1	8.6 ± 0.6	198.4 ± 33.1	
			Danuglipron, 50 mg bid	10	60.2 ± 7.97	50	9.1 ± 5.1	87.8 ± 13.8	32.6 ± 3.6	8.3 ± 0.9	168.1 ± 34.9	
			Danuglipron, 70 mg bid	9	58.3 ± 5.32	44.4	11.6 ± 4.6	86.9 ± 18.6	31.9 ± 4.3	8.3 ± 0.6	186.3 ± 32.0	
			Danuglipron, 120 mg bid	9	55.8 ± 7.68	77.8	10.8 ± 4.7	101.6 ± 17.4	35.0 ± 4.7	8.5 ± 1.0	196.9 ± 26.1	
			placebo	25	57.6 ± 7.71	48	8.5 ± 6.8	94.3 ± 17.7	33.3 ± 4.5	8.0 ± 0.8	167.6 ± 32.5	
Ono,2023	RCT, multicentre, double-blind	type 2 diabetes, obesity	Danuglipron, 40 mg bid	10	55.9 ± 10.0	80	2.9 ± 2.2	73.3 ± 9.9	28.6 ± 4.1	8.2 ± 1.1	173.3 ± 29.7	56 days
			Danuglipron, 80 mg bid	10	58.0 ± 6.7	88.9	9.1 ± 5.8	79。7 ± 13.6	28.2 ± 3.6	8.6 ± 1.0	175.7 ± 32.7	
			Danuglipron, 120 mg bid	9	50.7 ± 7.5	88.9	5.7 ± 7.7	79.6 ± 10.0	28.6 ± 3.4	8.4 ± 1.2	163.7 ± 47.5	
			placebo	9	58.6 ± 8.8	88.9	5.5 ± 4.1	81.5 ± 10.4	25.9 ± 2.7	8.3 ± 1.2	182.9 ± 36.3	
Saxena,2023	RCT, multicentre, double-blind	type 2 diabetes, obesity	Danuglipron, 2.5 mg bid	68	58.9 ± 9.30	56	8.8 ± 6.31	90.9 ± 20.13	32.5 ± 5.17	8.10 ± 1.03	169.3 ± 42.4	16 weeks
			Danuglipron, 10 mg bid	68	58.1 ± 9.43	51	8.5 ± 6.85	92.3 ± 16.44	33.0 ± 5.34	8.01 ± 0.91	165.4 ± 39.08	
			Danuglipron, 40 mg bid	71	59.6 ± 8.58	48	8.0 ± 5.82	90.2 ± 18.74	32.3 ± 5.25	8.00 ± 0.89	166.0 ± 39.33	
			Danuglipron, 80 mg bid	67	58.4 ± 9.18	52	9.7 ± 6.2	91.3 ± 16.64	32.9 ± 5.06	8.07 ± 0.95	172.8 ± 45.47	
			Danuglipron, 120 mg bid	71	58.8 ± 9.43	48	8.7 ± 7.89	93.1 ± 17.95	33.3 ± 5.70	8.05 ± 0.86	169.5 ± 40.65	
			placebo	66	57.9 ± 10.27	50	8.8 ± 6.90	90.1 ± 17.54	32.5 ± 5.08	8.24 ± 0.90	173.0 ± 43.74	
Saxena,2023	RCT, multicentre, double-blind	type 2 diabetes	Danuglipron, 80 mg bid LS	20	59.5 ± 9.55	50	9.33 ± 5.16	96.410 ± 20.43	35.1 ± 5.96	8.14 ± 1.025	178.3 ± 38.01	12 weeks
			Danuglipron, 80 mg bid HS	22	60.9 ± 8.69	54.5	10.38 ± 6.44	91.111 ± 14.22	32.8 ± 5.51	8.25 ± 1.019	177.5 ± 44.34	
			Danuglipron, 120 mg bid LF	22	58.3 ± 7.11	54.5	9.15 ± 5.78	101.880 ± 24.54	35.1 ± 6.89	8.05 ± 0.880	159.8 ± 48.52	
			Danuglipron, 120 mg bid HF	22	57.2 ± 11.80	54.5	10.20 ± 9.88	95.152 ± 17.01	33.9 ± 4.47	8.56 ± 1.162	182.9 ± 57.50	
			Danuglipron, 200 mg bid HF	21	59.0 ± 9.31	52.4	7.87 ± 4.83	86.365 ± 13.64	31.5 ± 3.76	8.24 ± 1.162	167.7 ± 53.90	
			placebo	16	53.9 ± 9.10	50	8.15 ± 7.58	101.017 ± 18.44	35.5 ± 5.93	7.83 ± 0.936	162.6 ± 44.68	
		obesity without diabetes	Danuglipron, 200 mg bid HF	22	48.5 ± 13.12	27.3	NA	103439 ± 12.72	37.4 ± 4.55	5.41 ± 0.413	95.8 ± 8.22	
			placebo	6	49.5 ± 5.79	33.3	NA	103.775 ± 15.76	36.7 ± 1.55	5.77 ± 0.258	104.5 ± 11.40	
Pratt,2023	RCT, multicentre, double-blind		Orforglipron, 9 mg qd	9	57.7 ± 6.4	44.4	13.48 ± 8.29	85.61 ± 12.76	30.14 ± 3.60	8.02 ± 0.62		12 weeks
			Orforglipron, 15 mg qd	10	59.6 ± 4.6	70	15.02 ± 11.97	88.02 ± 14.36	30.39 ± 3.61	7.84 ± 0.74		
			Orforglipron, 21 mg qd	14	55.3 ± 8.0	71.4	9.48 ± 5.48	92.09 ± 18.78	32.60 ± 5.48	8.36 ± 1.31		
			Orforglipron, 27 mg qd	9	58.8 ± 4.6	77.8	7.60 ± 4.39	92.80 ± 15.36	30.62 ± 3.55	7.82 ± 0.69		
			Orforglipron, 45 mg qd	9	62.8 ± 4.4	44.4	10.38 ± 4.78	81.49 ± 10.24	29.82 ± 2.84	7.93 ± 0.79		
			placebo	17	56.0 ± 6.0	58.8	8.63 ± 4.89	90.29 ± 20.04	31.31 ± 4.86	8.09 ± 0.75		
Pratt,2023	RCT, single centre, double-blind	obesity without diabetes	Orforglipron, 2 mg qd	9	42.5	73	NA	84	28.5	NA	84.85	4 weeks
			Orforglipron, 6 mg qd	9	42.5	73	NA	84	28.5	NA	83.79	
			Orforglipron, 16 mg qd	9	42.5	73	NA	84	28.5	NA	80.35	
			Orforglipron, 24 mg qd	18	42.5	73	NA	84	28.5	NA	85	
			placebo	15	42.5	73	NA	84	28.5	NA	87.42	
Wharton,2023	RCT, multicentre, double-blind	obesity without diabetes	Orforglipron, 12 mg qd	50	49.8 ± 10.5	38	NA	107.5 ± 25.3	37.7 ± 7.7	5.5 ± 0.4	94.4 ± 9.8	36 weeks
			Orforglipron, 24 mg qd	53	57.0 ± 9.1	43	NA	112.1 ± 30.2	38.1 ± 7.7	5.7 ± 0.3	97.5 ± 12.0	
			Orforglipron, 36 mg qd	58	55.9 ± 11.3	38	NA	108.3 ± 25.5	38.0 ± 6.3	5.7 ± 0.4	96.9 ± 13.3	
			Orforglipron, 45 mg qd	61	53.8 ± 11.9	43	NA	108.0 ± 24.4	37.7 ± 6.6	5.7 ± 0.4	95.2 ± 9.7	
			placebo	50	54.0 ± 8.8	42	NA	107.6 ± 25.2	38.7 ± 7.6	5.6 ± 0.4	97.2 ± 10.2	
Frias,2024	RCT, multicentre, double-blind	type 2 diabetes	Orforglipron, 3 mg qd	51	59.0 ± 9.4	51	5	99.3 ± 25.4	35.3 ± 8.2	8.0 ± 0.8	164.0 ± 40.9	26 weeks
			Orforglipron, 12 mg qd	56	57.4 ± 9.2	64	7.1	99.3 ± 18.1	34.8 ± 6.3	8.2 ± 0.9	172.1 ± 42.8	
			Orforglipron, 24 mg qd	47	60.5 ± 9.1	64	5.9	98.5 ± 22.9	34.1 ± 7.7	8.2 ± 0.9	171.7 ± 44.4	
			Orforglipron, 36 mg qd	61	59.7 ± 9.2	59	5.9	98.9 ± 17.5	34.4 ± 5.4	8.0 ± 0.7	157.9 ± 28.7	
			Orforglipron, 45 mg qd	63	58.5 ± 9.4	63	6.8	104.6 ± 25.1	36.4 ± 6.9	8.1 ± 0.9	166.4 ± 35.0	
			Danuglipron, 1.5 mg bid	50	58.8 ± 10.2	60	7.9	98.8 ± 22.1	35.4 ± 8.0	8.0 ± 0.7	167.6 ± 38.0	
			placebo	55	58.3 ± 9.5	51	7.8	102.0 ± 18.8	35.8 ± 6.2	8.1 ± 0.9	172.0 ± 42.9	

NA: not applicable.

According to the Cochrane Collaboration’s risk of bias tool, all included RCTs demonstrated low overall and domain-specific risks of bias (**Supplementary Figures 1**, **2**).

### 3.2 Glycated haemoglobin evaluation

**Figure 2:**
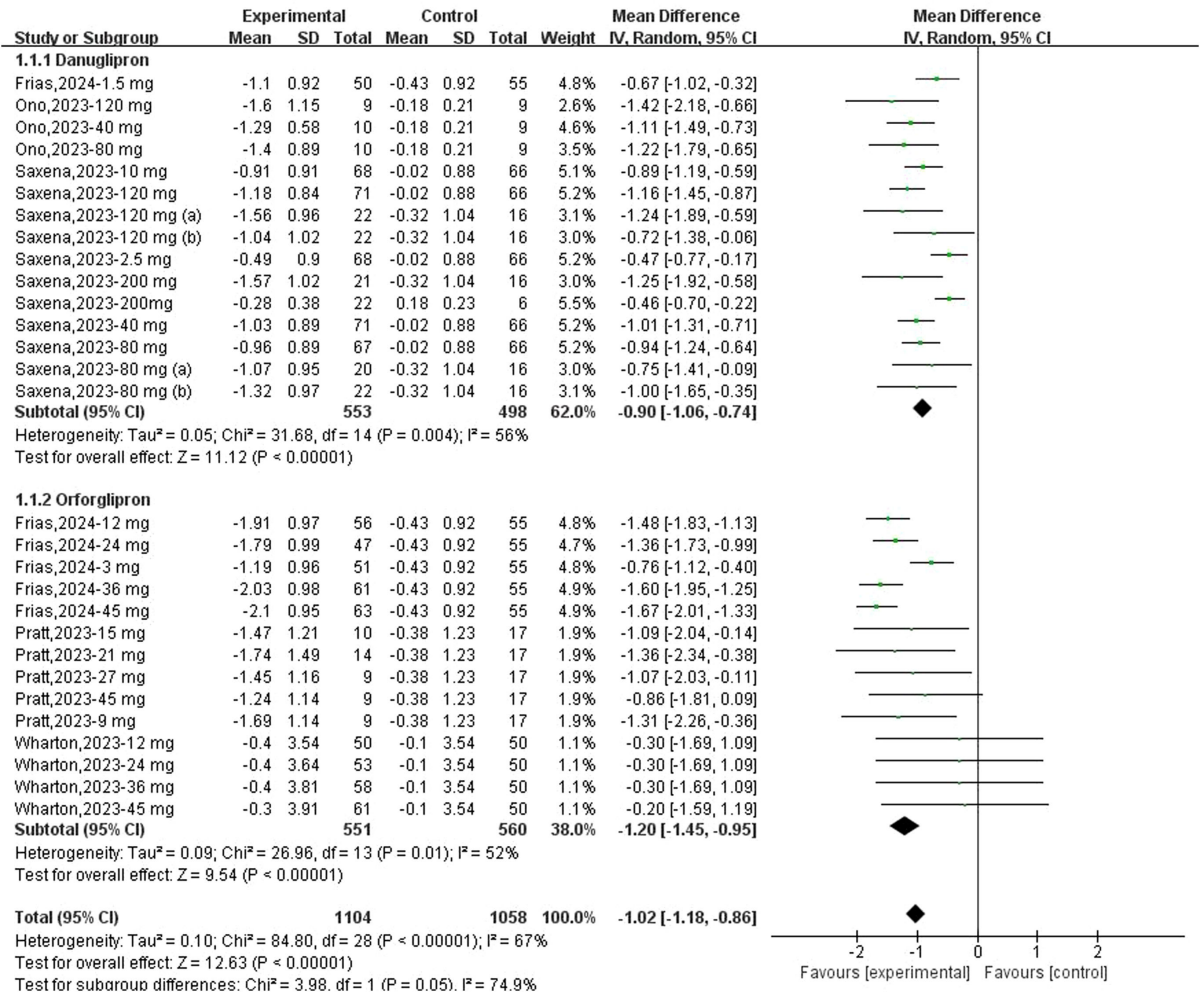
Forest plot of change of HbA1c (%) from baseline following danuglipron and orforglipron.

A total of 15 datasets were available to evaluate changes in HbA1c from baseline following danuglipron treatment. Heterogeneity assessment indicated significant inter-study variability (*I*² = 56%, *P* = 0.004), prompting the use of a random-effects model for pooled effect estimation. Compared with controls, danuglipron significantly reduced HbA1c from baseline (MD: −0.90%; 95% CI: −1.06, −0.74). Regarding orforglipron, 14 datasets were analysed for HbA1c changes. Heterogeneity was also observed (*I*² = 52%, *P* = 0.01), and the random-effects model demonstrated superior HbA1c reduction with orforglipron versus placebo (MD: −1.02%; 95% CI: −1.18, −0.86) (**Figure 2**).

Sensitivity analyses using sequential exclusion of individual studies were performed. For danuglipron, exclusion of one study (18) reduced heterogeneity to 34%, but the association with HbA1c reduction remained statistically significant (MD: −0.93%; 95% CI: −1.04, −0.82). Similarly, excluding one study (21) in the orforglipron analysis lowered heterogeneity to 26%, with sustained significant HbA1c improvement (MD: −1.42%; 95% CI: −1.57, −1.26).

### 3.3 Fasting plasma glucose and fasting plasma insulin evaluation

**Figure 3:**
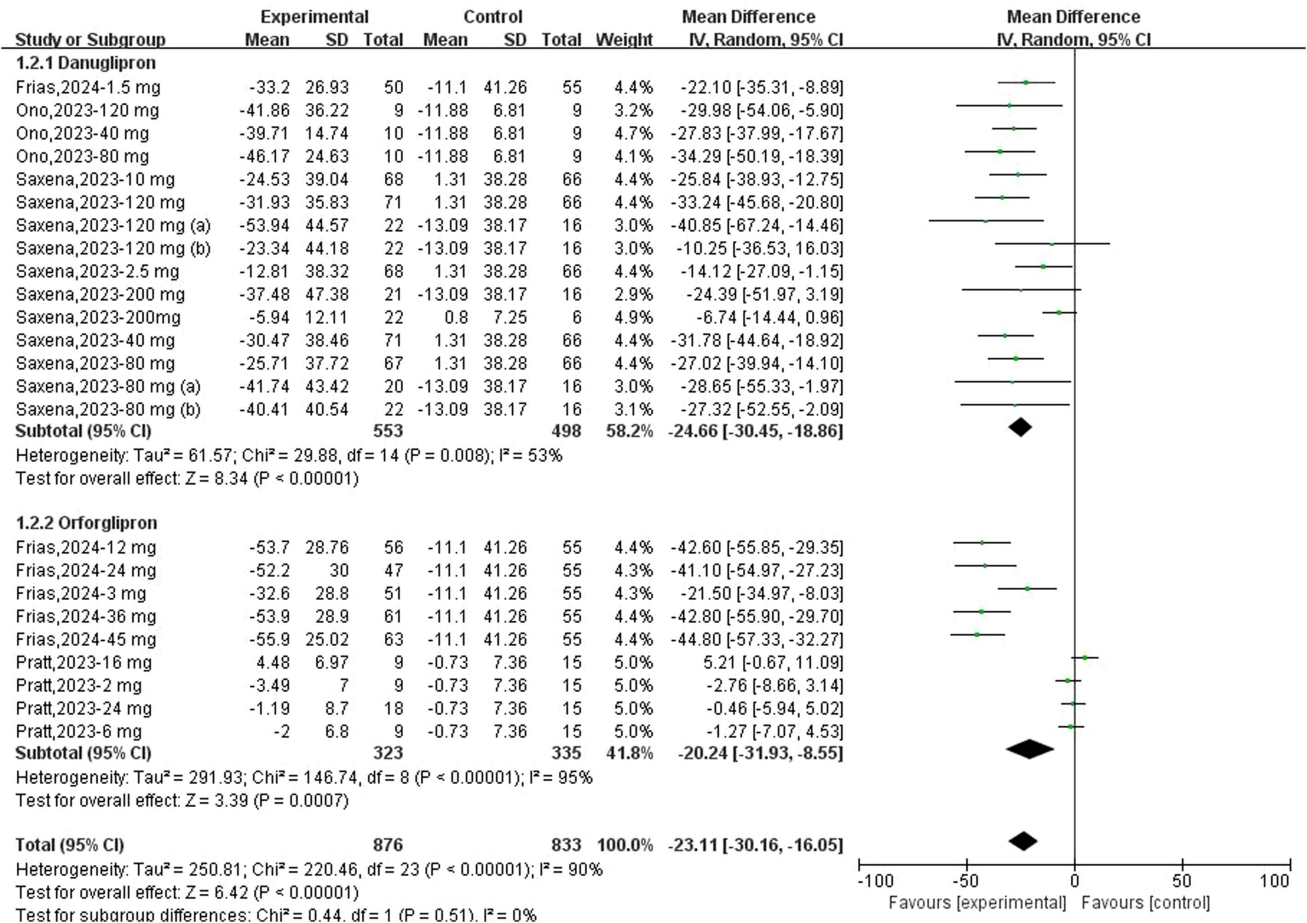
Forest plot of change of FPG (mg/dl) from baseline following danuglipron and orforglipron.

Fifteen datasets reported changes in FPG from baseline following danuglipron treatment. Heterogeneity assessment revealed significant inter-study variation (*I*² = 53%, *P* = 0.008), necessitating a random-effects model for pooled effect estimation. Meta-analysis demonstrated a significant reduction in FPG with danuglipron (MD: −24.66 mg/dL; 95% CI: −30.45, −18.86). Regarding orforglipron, nine datasets were analysed for FPG outcomes. Extreme heterogeneity (*I*² = 95%, *P* < 0.00001) justified the use of a random-effects model, which revealed a statistically significant association between orforglipron and FPG reduction compared with placebo (MD: −20.24 mg/dL; 95% CI: −31.93, −8.55) (**Figure 3**).

Sensitivity analyses involving sequential exclusion of individual studies were conducted. For danuglipron, removal of one study (18) eliminated heterogeneity (*I*² = 0%), but the FPG-lowering effect remained significant (MD: −26.91 mg/dL; 95% CI: −31.05, −22.78). In contrast, sensitivity analyses for orforglipron did not substantially reduce heterogeneity; however, the association between orforglipron and FPG reduction persisted, with statistical significance.

**Figure 4:**
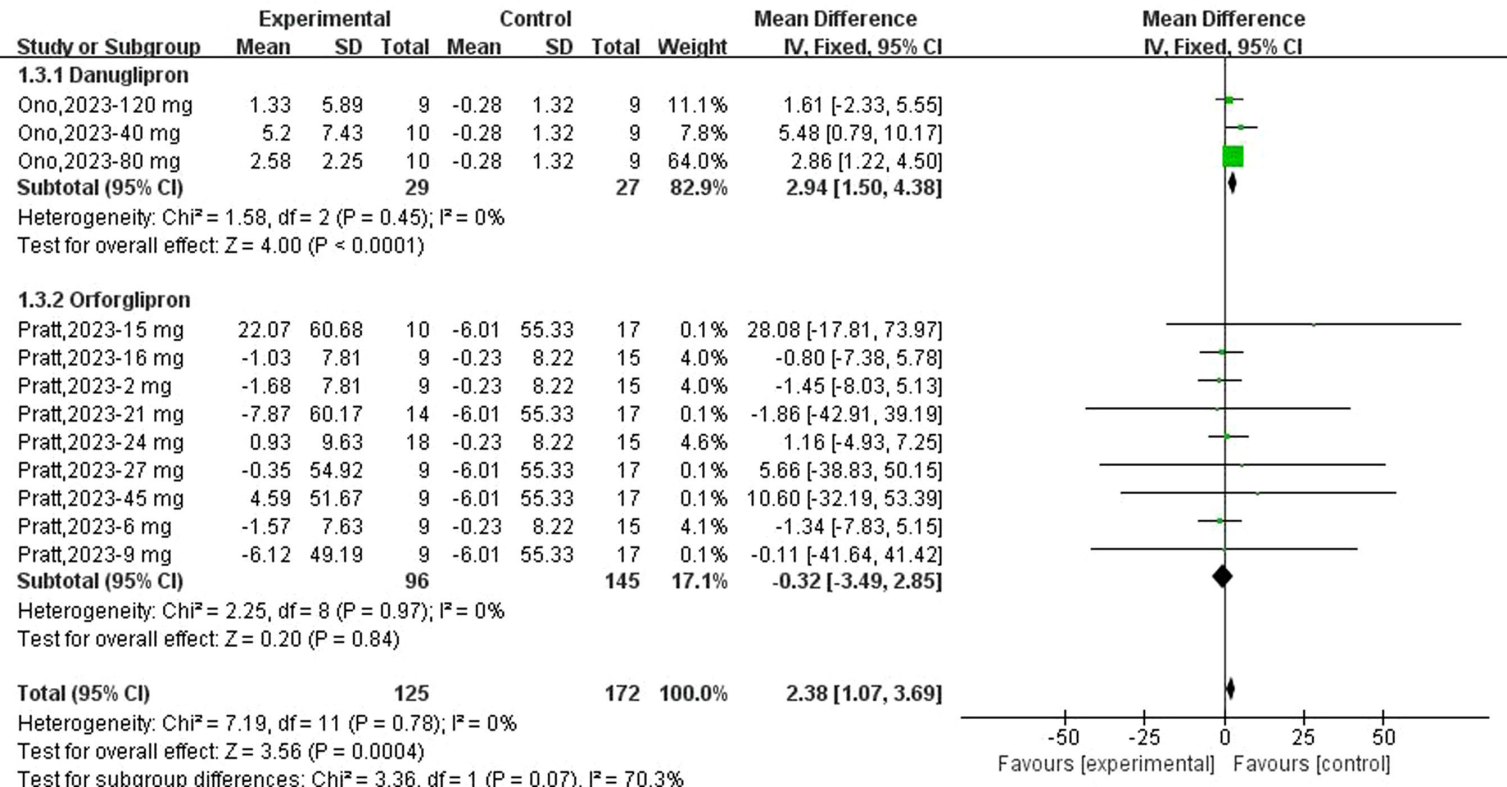
Forest plot of change of FPI (μIU/mL) from baseline following danuglipron and orforglipron.

Three datasets reported changes in FPI from baseline following danuglipron treatment. Heterogeneity assessment indicated no significant inter-study variability (*I*² = 0%, *P* = 0.45), and a fixed-effects model was applied for pooled effect estimation. Meta-analysis revealed a significant increase in FPI with danuglipron (MD: +2.94 μIU/mL; 95% CI: 1.50, 4.38). Regarding orforglipron, nine datasets were analysed to evaluate its effect on FPI. Heterogeneity analysis demonstrated homogeneity across studies (*I*² = 0%, *P* = 0.97), justifying the use of a fixed-effects model. The meta-analysis found no statistically significant association between orforglipron and FPI changes compared with placebo (MD: −0.32 μIU/mL; 95% CI: −3.49, 2.85) (**Figure 4**).

### 3.4 Body weight and body mass index

**Figure 5:**
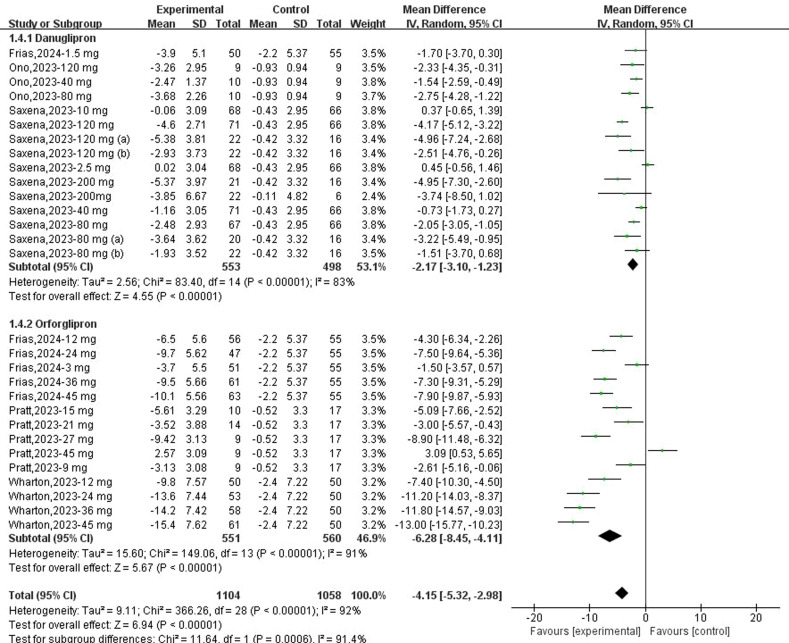
Forest plot of change of body weight from baseline following danuglipron and orforglipron.

**Figure 6:**
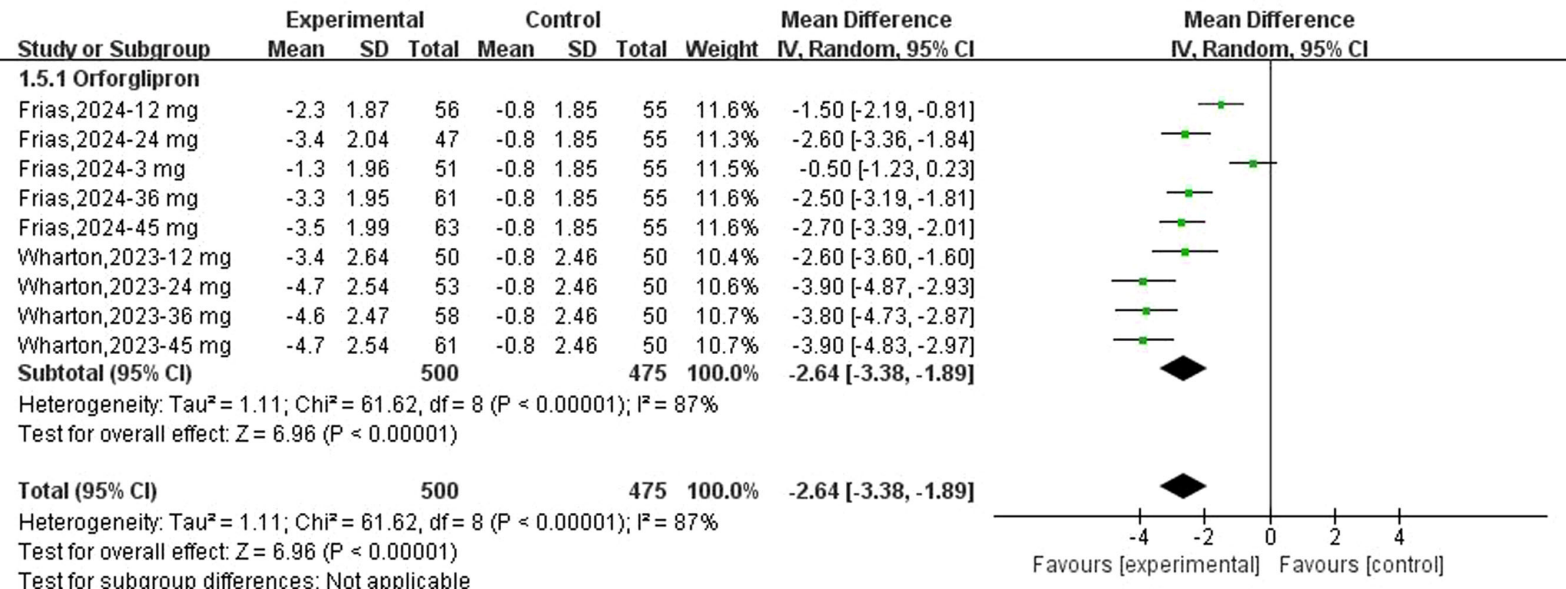
Forest plot of change of BMI from baseline following orforglipron.

Fifteen datasets reported changes in body weight from baseline following danuglipron treatment. Heterogeneity assessment revealed substantial inter-study variability (*I*² = 83%, *P* < 0.00001), prompting the use of a random-effects model. Meta-analysis demonstrated a statistically significant reduction in body weight with danuglipron (MD: −2.17 kg; 95% CI: −3.10, −1.23). Regarding orforglipron, 14 datasets were analysed to evaluate weight-related outcomes. Extreme heterogeneity (*I*² = 91%, *P* < 0.00001) justified the random-effects model, which showed a pronounced weight reduction with orforglipron compared with placebo (MD: −6.28 kg; 95% CI: −8.45, −4.11) (**Figure 5**). Furthermore, the analysis of BMI changes (MD: −2.64 kg/m²; 95% CI: −3.38, −1.89) (**Figure 6**) corroborated orforglipron’s robust efficacy in weight management. Sensitivity analyses involving sequential exclusion of individual studies failed to identify significant sources of heterogeneity for either weight or BMI outcomes. Despite this, the associations between both danuglipron and orforglipron and improvements in weight and BMI remained statistically significant.

### 3.5 Safety

**Figure 7:**
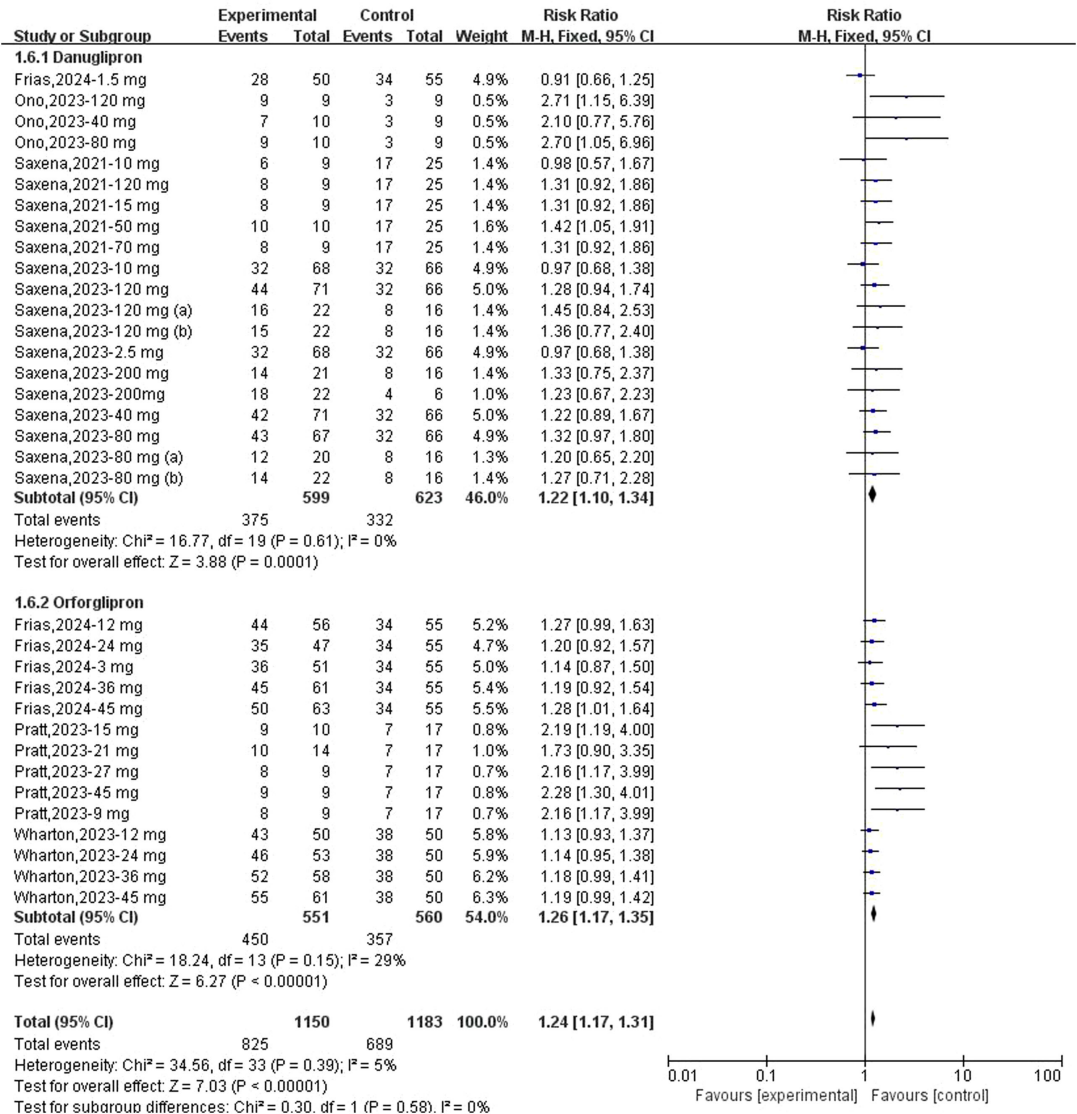
Forest plot of any TEAE incidence risk following danuglipron and orforglipron.

**Figure 8:**
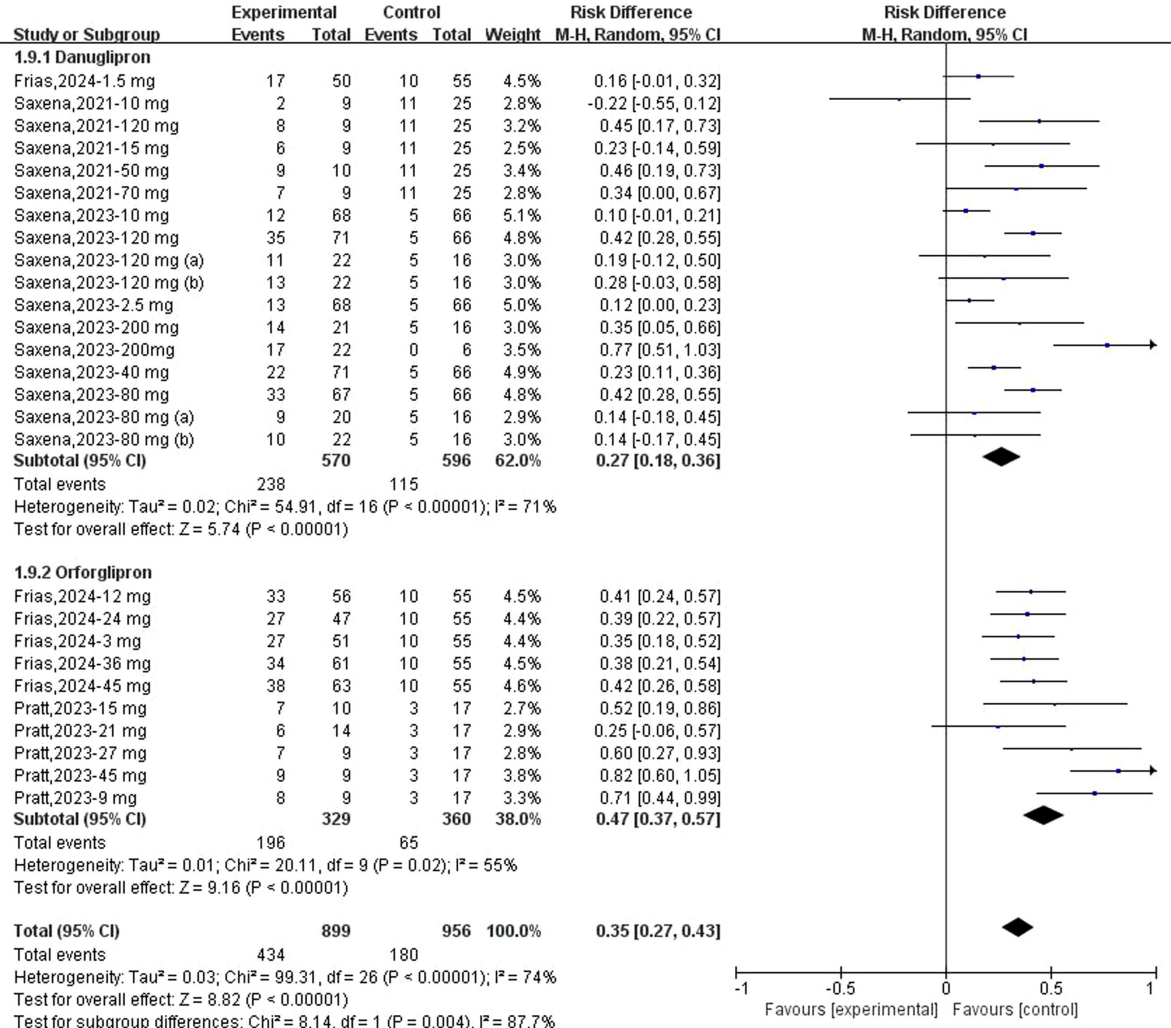
Forest plot of GI AE incidence risk following danuglipron and orforglipron.

Regarding the association between danuglipron/orforglipron and TEAEs, meta-analyses demonstrated a statistically significant increase in TEAE risk for both danuglipron (RR: 1.22; 95% CI: 1.10–1.34; *I*² = 0%) and orforglipron (RR: 1.26; 95% CI: 1.17–1.35; *I*² = 29%) (**Figure 7**). Additionally, both agents were significantly associated with gastrointestinal AEs: danuglipron (RD: 0.27; 95% CI: 0.18–0.36; *I*² = 71%) and orforglipron (RD: 0.47; 95% CI: 0.37–0.57; *I*² = 55%) (**Figure 8**). Subgroup analyses revealed elevated risks for specific gastrointestinal symptoms, including diarrhoea (danuglipron: RD: 0.06; 95% CI: 0.03–0.09; orforglipron: RD: 0.10; 95% CI: 0.06–0.14) (**Supplementary Figure 3**), dyspepsia (danuglipron: RD: 0.06; 95% CI: 0.04–0.09; orforglipron: RD: 0.04; 95% CI: 0.01–0.07) (**Supplementary Figure 4**), nausea (danuglipron: RD: 0.26; 95% CI: 0.26–0.35; orforglipron: RD: 0.33; 95% CI: 0.26–0.39) (**Supplementary Figure 5**), constipation (danuglipron: RD: 0.04; 95% CI: −0.01–0.09; orforglipron: RD: 0.15; 95% CI: 0.11–0.19) (**Supplementary Figure 6**) and vomiting (danuglipron: RD: 0.17; 95% CI: 0.09–0.25; orforglipron: RD: 0.25; 95% CI: 0.17–0.32) (**Supplementary Figure 7**).

In addition, a GRADE assessment indicated high certainty of evidence for the main efficacy and safety outcomes (**Supplementary Table S1**). Exploratory dose–response analyses suggested a non-significant trend towards greater HbA1c reduction with increasing doses (*P* = 0.181) (**Supplementary Figure 8**).

## 4 Discussion

In this meta-analysis of prospective studies, we systematically evaluated the effects of novel oral GLP-1 RAs, danuglipron and orforglipron, on glycaemic control and weight management in patients with T2DM and obesity compared with placebo, as well as comprehensively assessing their impacts on AEs, particularly gastrointestinal AEs. Our findings align with previous small-scale studies (22, 23), and this updated analysis provides robust evidence that not only adheres to biological plausibility but also expands clinical understanding through comprehensive quantitative synthesis, offering valuable insights for guiding the clinical use of these novel oral GLP-1 RAs.

In recent years, the GLP-1 R has emerged as a pivotal therapeutic target for T2DM. Although injectable GLP-1 RAs have demonstrated glycaemic benefits clinically, the development of oral formulations remains imperative to overcome limitations associated with injectable administration. This systematic review and meta-analysis synthesised evidence from eight rigorously conducted RCTs, indicating that both danuglipron and orforglipron achieve significant improvements in glycaemic parameters among patients with T2DM and obesity. The marked reductions in HbA1c (danuglipron: MD: −0.90%; orforglipron: MD: −1.02%) and FPG (danuglipron: MD: −24.66 mg/dL; orforglipron: MD: −26.91 mg/dL) are consistent with the established pharmacodynamic actions of GLP-1 RAs, which enhance glucose-dependent insulin secretion via cAMP signalling pathways and suppress α-cell glucagon release (24, 25). According to recent meta-analytic evidence, 7 and 14 mg of semaglutide once daily reduced HbA1c by 1.06% and 1.1%, respectively, compared with placebo in patients with T2DM (26). Furthermore, the same doses of semaglutide demonstrated superior HbA1c reductions of 0.26% and 0.38%, respectively, versus other glucose-lowering agents (26). The divergent effects on FPI (danuglipron increased FPI [MD + 2.94 μIU/mL] whereas orforglipron exhibited neutral effects) likely reflect differences in receptor binding affinities or tissue-specific activation patterns. Additionally, danuglipron’s FPI elevation may resemble early-phase insulinotropic effects observed with short-acting agents, raising concerns about its long-term implications for β-cell exhaustion.

Significant weight reduction was observed with both agents, particularly highlighting orforglipron’s superior efficacy in reducing body weight (MD: −6.28 kg) and BMI (MD: −2.64 kg/m²), underscoring their dual role in metabolic regulation. Previous studies reported that 7 mg of semaglutide induced a weight loss of 1.18 kg versus placebo and 1.47 kg compared with other glucose-lowering agents (26). However, the lack of FPI improvement with orforglipron warrants caution, as suppressed insulin levels in the context of weight loss may paradoxically exacerbate dyslipidaemia in susceptible individuals – a phenomenon observed in certain lifestyle intervention trials. Clinicians should therefore balance the benefits of weight reduction against potential metabolic trade-offs, particularly in patients with comorbid cardiovascular disease (27).

The safety profiles of both agents were dominated by gastrointestinal AEs, such as nausea and vomiting, consistent with the class effects of GLP-1 RAs. Mechanistically, these AEs arise from delayed gastric emptying and direct stimulation of GLP-1 Rs in the area postrema, a circumventricular organ mediating emesis. For danuglipron, gastrointestinal side effects may be target mediated, and the presence of a carboxylic acid moiety in its structure could contribute to these outcomes by influencing its pharmacokinetic properties, dosing regimen and direct gastrointestinal irritation (28). The incidence of danuglipron-related AEs exhibits dose dependency. Although Phase 1 trial data suggested no dose-dependent increase in gastrointestinal AEs (15), another Phase 1 trial observed the lowest AE rates at low doses (10–15 mg), with 100% AE incidence at 120 mg, indicating a clear dose–response relationship for gastrointestinal tolerability (14). Furthermore, danuglipron’s Phase 2 study demonstrated escalating discontinuation rates and AE frequencies as the target dose increased from 80 mg twice daily to 200 mg twice daily (18). Regarding orforglipron, most AE risks appear linked to rapid dose escalation and were more pronounced in aggressive titration groups (21). However, its Phase 1b trial revealed no significant dose-dependent effects, potentially due to the limited sample size and short exposure duration (17). Emerging pharmacogenomic evidence suggests that polymorphisms in the GLP-1 R gene (*GLP1R*) may modulate AE susceptibility, explaining interpatient variability. For example, carriers of the rs6923761 G-allele exhibit attenuated gastrointestinal responses to GLP-1 RAs, a finding that merits integration into personalised treatment algorithms (29, 30). Future trials should evaluate whether intermittent dosing or adjunctive antiemetics can mitigate AE-related discontinuations without compromising therapeutic efficacy.

We observed a modest increase in FPI among participants treated with danuglipron in our pooled analysis. Several non-mutually exclusive mechanisms may explain this finding. First, GLP-1 R agonism augments glucose-dependent insulin secretion, and early improvements in β-cell responsiveness could manifest as higher fasting insulin levels, particularly in short-term trials (31). Second, changes in hepatic insulin clearance or transient compensatory hyperinsulinemia related to concurrent background therapies (e.g. use of insulin secretagogues or withdrawal/alteration of other agents) might increase measured fasting insulin independently of insulin sensitivity (32). Third, methodological factors – including heterogeneity in assay methods, sampling timing, the relatively short duration of most included trials and limited sample sizes – could have contributed to an apparent rise in FPI (33). Importantly, the clinical importance of this short-term FPI increase is uncertain – an isolated rise in fasting insulin does not necessarily indicate progressive β-cell dysfunction and may reflect transient adaptations (34).

A contemporary and closely related meta-analysis by Karakasis et al. (35) examined randomised trials (n = 1,037) of oral small-molecule GLP-1 RAs (danuglipron and orforglipron) and reported broadly similar effects on glycaemia and body weight (HbA1c reduction = −1.03%, FPG = −28.5 mg/dL, weight = −4.3 kg), as well as an increased incidence of gastrointestinal AEs. Notably, Karakasis et al. complemented their synthesis with a dose–response meta-analysis using restricted cubic splines along with a GRADE assessment of evidence certainty, which provided useful insight into dose–effect relationships and the strength of available evidence. The present analysis is consistent with their principal findings; importantly, however, our study extends the evidence base by including more recent trials and a larger pooled sample (n = 1,454 in our review) and by reporting additional outcomes (e.g. fasting insulin and a more granular safety analysis). Although Karakasis et al. provided formal dose–response curves and GRADE ratings, these analyses were not included in the present manuscript due to differences in available dose-level reporting across the newly added trials. Nevertheless, the concordance between the two independent meta-analyses strengthens confidence in the emerging clinical profile of these oral GLP-1 RAs. We acknowledge that incorporating formal GRADE and dose–response meta-analyses into future revisions (or as supplementary analyses) would further increase the interpretability and clinical applicability of the results.

The findings suggest that oral small-molecule GLP-1 RAs achieve the class’s expected metabolic benefits (HbA1c and weight reduction) while offering the practical advantage of oral dosing, which may improve uptake and adherence among patients unwilling or unable to use injectables. However, several factors limit immediate translation into routine care. Large, long-term outcome data – including cardiovascular safety and durability of glycaemic/weight effects – are lacking for these oral agents, and head-to-head comparisons with established injectable GLP-1 RAs are not yet available. Tolerability concerns (notably gastrointestinal AEs) and a modest short-term signal of increased fasting insulin also warrant cautious interpretation and monitoring. To define the optimal place of these agents in clinical pathways, future priorities should include independent Phase 3 trials, pragmatic real-world studies, direct comparative trials against injectable GLP-1 RAs, standardised reporting of concomitant glucose-lowering therapies and metabolic dynamic measures, and formal cost-effectiveness and adherence evaluations. Until such evidence accrues, clinicians should weigh the convenience of oral therapy against unresolved questions regarding long-term safety, comparative effectiveness and patient selection.

Several limitations affect the interpretability of these findings. First, as most included studies were early-phase clinical trials, the pooled sample size (n = 1,454) and short trial durations (≤36 weeks) preclude definitive conclusions regarding rare safety outcomes or the durability of metabolic benefits. Second, considerable heterogeneity was observed in weight and fasting glucose outcomes; because the included trials were generally small and underpowered for detailed subgroup analyses, the sources of this heterogeneity could not be fully resolved. Third, heterogeneity in background therapies across trials – particularly variable use of SGLT2 inhibitors and metformin – may have confounded the observed treatment effects, but insufficient and inconsistently reported data precluded further exploration of these confounders’ potential impacts. Finally, all included trials were industry sponsored, which may introduce reporting or publication bias. Future research should prioritise large, independently funded Phase 3 trials, incorporate real-world evidence to evaluate these agents in underrepresented populations (e.g. advanced chronic kidney disease) and conduct cost-effectiveness analyses to inform healthcare resource allocation.

## 5 Conclusions

This study’s findings suggest that the novel small-molecule GLP-1 RAs danuglipron and orforglipron represent effective and safe options for glycaemic management in patients with T2DM. Notably, both agents also demonstrate considerable benefits in weight control. While orforglipron and similar oral GLP-1RAs show promise, current evidence is insufficient to conclude that they are ready alternatives to established therapies. Large, long-duration RCTs with thorough safety monitoring and independent outcome adjudication are required to define the long-term efficacy, tolerability and risk–benefit profile of oral small-molecule GLP-1RAs.

## Data Availability

The original contributions presented in the study are included in the article/**Supplementary Material**. Further inquiries can be directed to the corresponding author.
