# Supplementary material for: The efficacy and safety of danuglipron and orforglipron in patients with type 2 diabetes and obesity: a systematic review and meta-analysis
Source: Front Endocrinol (Lausanne). 2025 Dec 10;16:1646956. doi: 10.3389/fendo.2025.1646956 (PMC12727569; doi:10.3389/fendo.2025.1646956)
Supplement: Supplementary file 1 [file DataSheet1.docx]

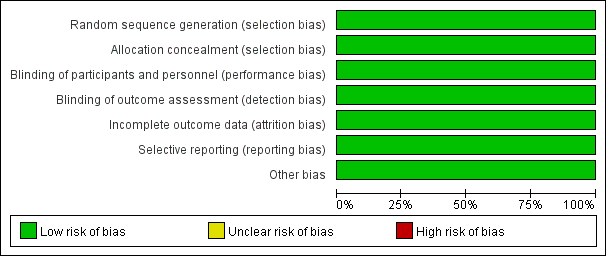


Supplementary Figure 1 Risk of bias graph


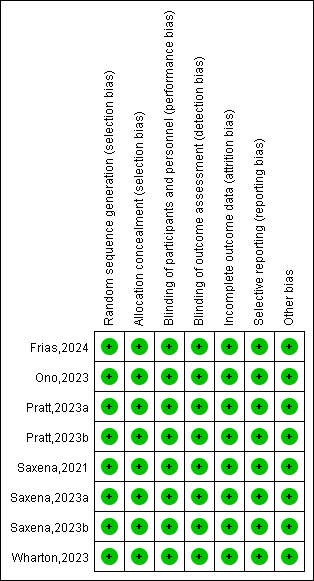


Supplementary Figure 2 Risk of bias summary


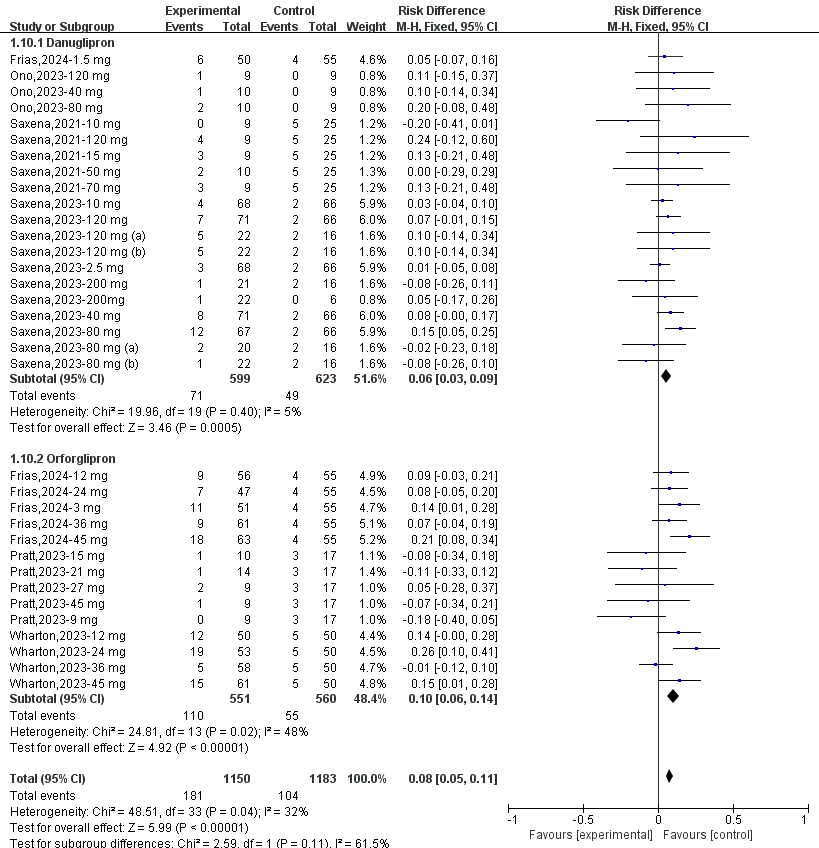


Supplementary Figure 3: Forest plot of diarrhea incidence risk following danuglipron or orforglipron


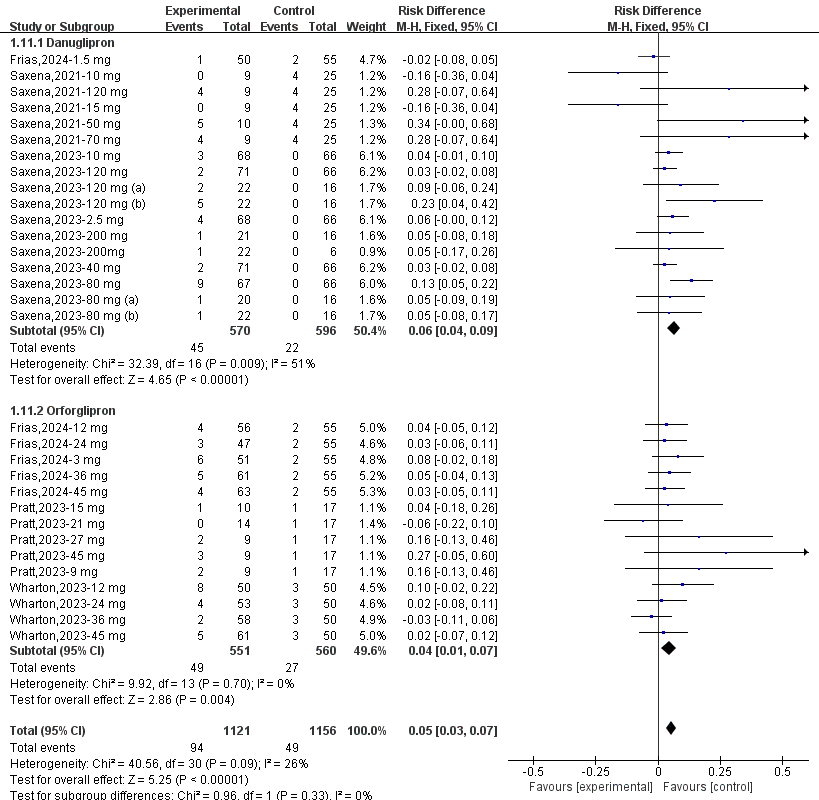


Supplementary Figure 4: Forest plot of dyspepsia incidence risk following danuglipron or orforglipron


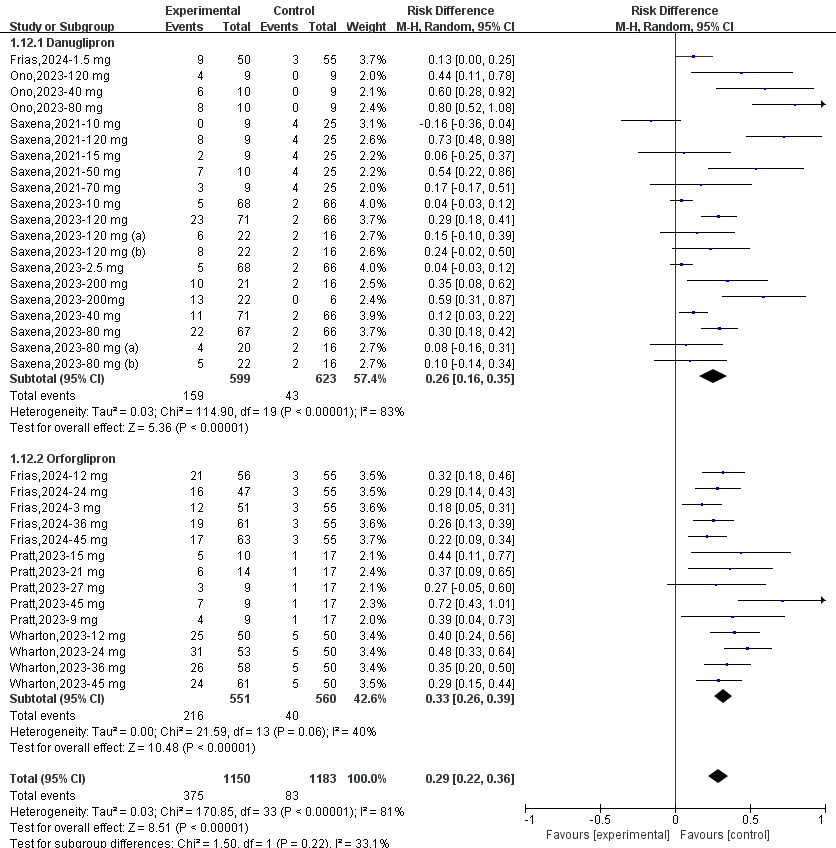


Supplementary Figure 5: Forest plot of nausea incidence risk following danuglipron or orforglipron


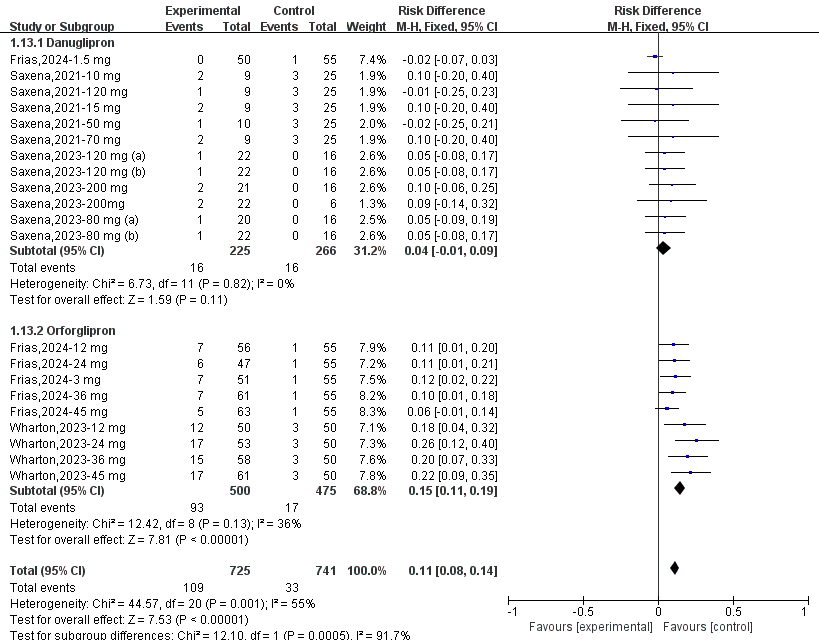


Supplementary Figure 6: Forest plot of constipation incidence risk following danuglipron or orforglipron


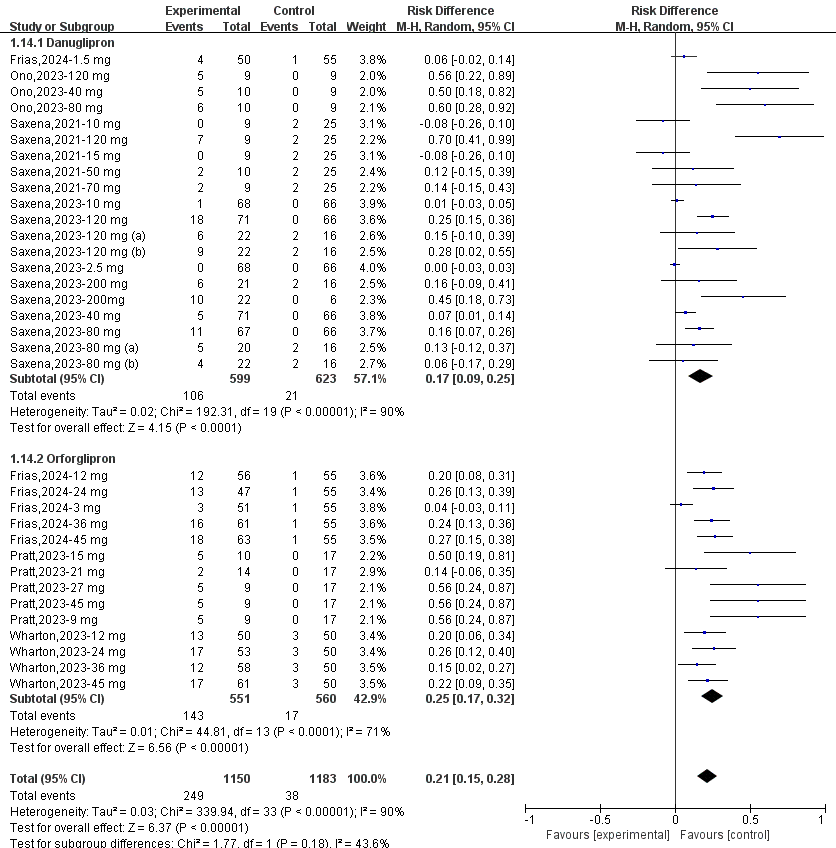


Supplementary Figure 7: Forest plot of vomiting incidence risk following danuglipron or orforglipron


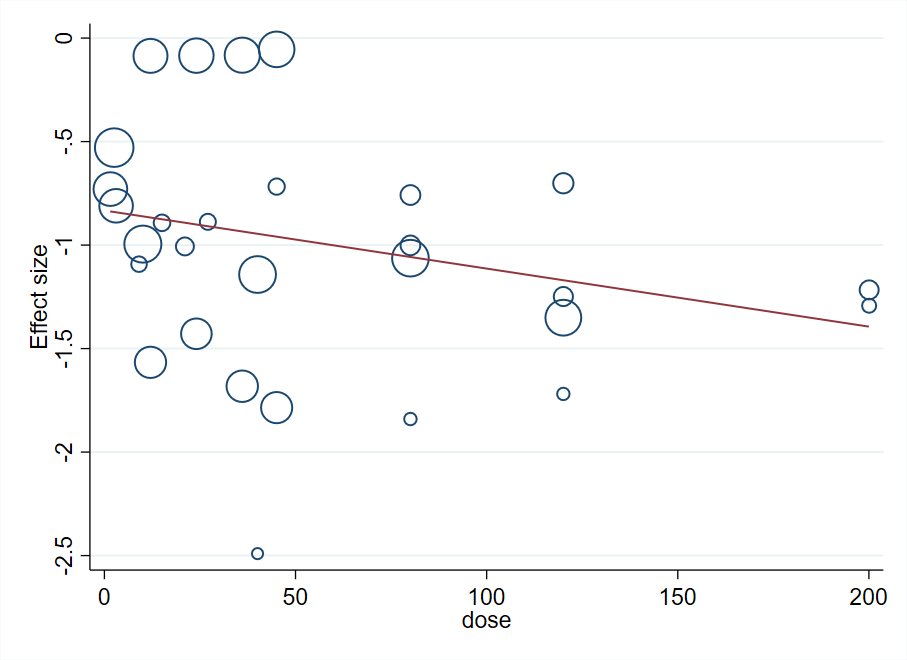


Supplementary Figure 8: Dose–response regression analyses for dose vs HbA1c
