# Supplementary material for: The efficacy and safety of danuglipron and orforglipron in patients with type 2 diabetes and obesity: a systematic review and meta-analysis
Source: Front Endocrinol (Lausanne). 2025 Dec 10;16:1646956. doi: 10.3389/fendo.2025.1646956 (PMC12727569; doi:10.3389/fendo.2025.1646956)
Supplement: Supplementary file 2 [file Table1.docx]

Table S1 GRADE evaluation for primary outcomes

| **Certainty assessment** | | | | | | | **№ of patients** | | **Effect** | | **Certainty** | **Importance** |
| --- | --- | --- | --- | --- | --- | --- | --- | --- | --- | --- | --- | --- |
| **№ of studies** | **Study design** | **Risk of bias** | **Inconsistency** | **Indirectness** | **Imprecision** | **Other considerations** | **[intervention]** | **[comparison]** | **Relative (95% CI)** | **Absolute (95% CI)** |  |  |
| **HbA1C** | | | | | | | | | | | | |
| 6 | randomised trials | not serious | not serious | not serious | not serious | none | 1104 | 1058 | - | MD **1.02 lower** (1.18 lower to 0.86 lower) | ⨁⨁⨁⨁ High |  |
| **FPG** | | | | | | | | | | | | |
| 5 | randomised trials | not serious | not serious | not serious | not serious | none | 876 | 833 | - | MD **23.11 lower** (30.16 lower to 16.05 lower) | ⨁⨁⨁⨁ High |  |
| **FPI** | | | | | | | | | | | | |
| 3 | randomised trials | not serious | not serious | not serious | not serious | none | 125 | 172 | - | MD **2.38 higher** (1.07 higher to 3.69 higher) | ⨁⨁⨁⨁ High |  |
| **weight** | | | | | | | | | | | | |
| 6 | randomised trials | not serious | not serious | not serious | not serious | none | 1104 | 1058 | - | MD **4.15 lower** (5.32 lower to 2.98 lower) | ⨁⨁⨁⨁ High |  |
| **BMI** | | | | | | | | | | | | |
| 2 | randomised trials | not serious | not serious | not serious | not serious | none | 500 | 475 | - | MD **2.64 lower** (3.38 lower to 1.89 lower) | ⨁⨁⨁⨁ High |  |
| **any AE** | | | | | | | | | | | | |
| 7 | randomised trials | not serious | not serious | not serious | not serious | none | 825/1150 (71.7%) | 689/1183 (58.2%) | **RR 1.24** (1.17 to 1.31) | **140 more per 1,000** (from 99 more to 181 more) | ⨁⨁⨁⨁ High |  |
| **GI AE** | | | | | | | | | | | | |
| 5 | randomised trials | not serious | not serious | not serious | not serious | none | 434/899 (48.3%) | 180/956 (18.8%) | **RR 0.35** (0.27 to 0.43) | **350 fewer per 1,000** (from 430 fewer to 270 fewer) | ⨁⨁⨁⨁ High |  |

**CI:** confidence interval; **MD:** mean difference; **RR:** risk ratio
